# An IgG4-Related Salivary Gland Disorder: A Case Series Presenting with a Different Clinical Setting

**DOI:** 10.1155/2011/236079

**Published:** 2011-07-19

**Authors:** Masayuki Ishida, Hiroaki Fushiki, Yukio Watanabe

**Affiliations:** Department of Otolaryngology, Head & Neck Surgery, University of Toyama, Toyama 930-0194, Japan

## Abstract

Küttner tumor is a chronic inflammatory disease that presents with a firm swelling of the submandibular gland and often mimics a neoplasm. Recently evidence suggests that Küttner tumor may be a type of disorder characterized by IgG4-related inflammations. Herein, we report 3 cases of submandibular gland swellings with severe fibrosis, inflammation with marked lymphoplasmacytic infiltration; this pathology mimics clinical manifestation of a malignant tumor in 18-fluorodeoxyglucose positron emission tomography (FDG-PET) findings.

## 1. Introduction

Küttner tumor, commonly known as chronic sclerosing sialadenitis, is a chronic inflammatory disease that presents with firm swelling of the submandibular gland and often mimics a malignant tumor clinically [1]. Recent studies have shown that the pathogenesis of Küttner tumor can be characterized by IgG4-related multifocal inflammations [2, 3]. In the present study, we report 3 cases of an IgG4-related salivary gland disorder that cause bilateral or unilateral swelling of the submandibular gland, each presenting with a different clinical setting. Diagnostic features of the disorder will be discussed together with a literature review.

## 2. Case Report

**Case 1 .**

**Figure 1:**
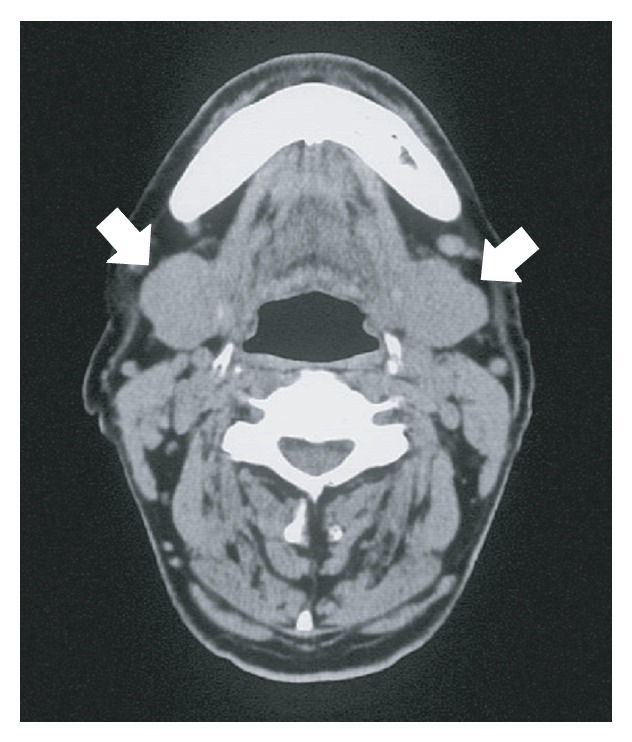
Cervical CT images of Case 1. Bilateral swelling of the submandibular glands (arrows).

A 77-year-old male visited our university hospital because of a 2-month history of bilateral swelling of the submandibular region. Bilateral diffused enlargement of the submandibular glands was elastic hard and had good mobility on palpation. A computed tomography (CT) scan showed that the submandibular gland was bilaterally enlarged to approximately 30 mm (Figure 1). There was no significant swelling of lymph nodes in the neck. Laboratory tests were negative for SS-A and SS-B antibodies, with no increase in amylase activity. The swelling could not be resolved by treatment with antibiotics and antiphlogistic agents. A biopsy sample of the left submandibular gland was taken under local anesthesia. The biopsied tissue was white and hard. Histopathological examination revealed extensive fibrosis and diffuse lymphocyte infiltration, with scattering of a small number of eosinophilic leucocytes and lymphoid follicles. The salivary gland tissue was highly atrophied.

**Case 2 .**

A 59-year-old female was admitted to our university hospital because of a swelling in the right submandibular gland region for the third time. Swellings in the right submandibular gland region had occurred twice in the previous 2 years, and the biopsy samples showed “reactive lymph node” both times. CT showed that the right submandibular gland was enlarged to 40 mm × 20 mm, with several lymph nodes measuring up to 15 mm in the periphery of the parotid gland and the right upper deep cervical region.

**Figure 2:**
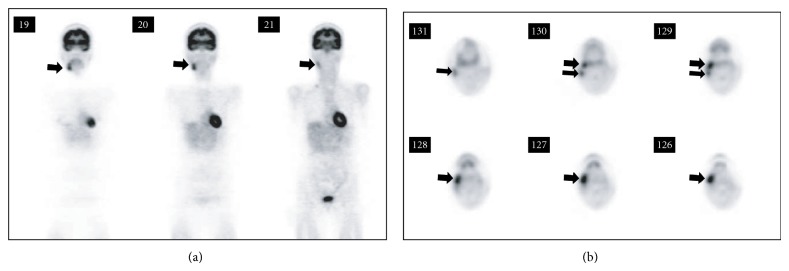
FDG-PET images of Case 2. (a) Intense uptake (SUV: 6.24) in the right submandibular gland (arrows). (b) Intense uptake in the right submandibular gland (thick arrows) and the deep interior cervical area (thin arrows).

**Figure 3:**
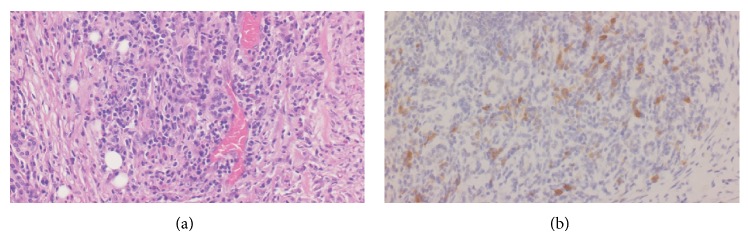
Histopathological examination of Case 2. (a) Small-sized gland ducts with plasma cell and eosinophilic leukocyte infiltrations (H&E stain  ×40). (b) IgG4-positive plasma cells (stained with IgG4 monoclonal antibody  ×40). Mouse monoclonal antibody to human IgG4 (clone, HP6025; dilution, 1 : 100; Zymed Laboratories Inc., Calif, USA).

18-fluorodeoxyglucose positron emission tomography (FDG-PET) showed a high and slightly heterogeneous increase in accumulations of FDG in the right submandibular region (standardized uptake value, SUV: 6.24), with a slight increase in accumulations in the upper dorsal to the right submandibular region, the right cervical regions, and the supraclavicular fossa (Figure 2). Laboratory tests were negative for SS-A and SS-B antibodies, with no increase in amylase activity. In order to rule out a possible right submandibular gland cancer with lymph node metastases or malignant lymphoma, the right submandibular gland was extirpated under general anesthesia. Histopathological examination revealed diffused, double-layered, small-sized gland ducts accompanied by lymphoid follicles forming a high-degree lymphocyte infiltration. Plasma cell and eosinophilic leukocyte infiltrations, relatively high-degree fibrosis, and many IgG4-positive plasma cells were also observed (Figure 3). Subsequent swelling occurred in the unaffected submandibular gland after a period, which was resolved by steroid treatment.

**Case 3 .**

A 61-year-old male was admitted to our university hospital because of a 1-year history of swelling of the right submandibular region. He reported that the swelling enlarged gradually over the year. CT showed a slightly contrast-enhanced mass, measuring 32 mm × 22 mm in the right submandibular region. Salivary gland scintigraphy (Tc-99 m) showed a decrease in accumulations in the right posterior submandibular gland in early images. Laboratory tests were negative for SS-A and SS-B antibodies, with no increase in amylase activity. The right submandibular gland was extirpated under general anesthesia. Histopathological examination revealed that the submandibular glands were enlarged because of marked lymphocyte infiltration and only a few normal submandibular glands were present. There was a remarkably high degree of plasma cell infiltration in the fibroid portion of the glands.

## 3. Discussion

In 1896, Küttner identified a disorder that causes tumor-like swelling of the submandibular glands, which was named after him [1]. It differs from nonspecific chronic sialadenitis in that the submandibular gland is commonly affected, hard tumor-like mass is present in clinical manifestation, and pathology shows florid lymphoplasmacytic infiltrate accompanied by prominent and progressive fibrosis. In 108 cases described by Isacsson and Lundquist, the mean age at diagnosis was 42–45 years, with no gender difference [4]. The chief complaint is recurrent pain and/or swelling in one side of the neck [4]. Recent studies have shown that Küttner tumor and Mikulicz's disease are serologically similar disorders that cause IgG4-related inflammations of the salivary gland and they both may produce similar lesions in organs other than the salivary gland [2, 3, 5–7]. Lesions of IgG4-related inflammations include the lacrimal glands, autoimmune pancreatitis, sclerosing cholangitis, interstitial nephritis, and retroperitoneal fibrosis [6, 7].

The 3 cases reported in this study are summarized in Table 1. A definite diagnosis of the disorders in this study was made by histopathological examination of the salivary glands; all cases were diagnosed with IgG4-related chronic sclerosing sialadenitis, on the basis of the presence of high-degree infiltration of IgG4-positive plasma cells in the swollen submandibular gland tissues. 

All 3 cases showed swelling of the submandibular region, but each presented with a different clinical manifestation. If the disease affects both sides, as in Case 1, systemic autoimmune disorders such as Küttner tumor, Mikulicz's disease, or Sjögren's syndrome should be considered initially in the differential diagnosis. Mikulicz's disease, first reported by Mikulicz in 1892, is a disorder of unknown cause, showing sustained left-right symmetric swellings of the lacrimal gland as well as the salivary gland [8]. In contrast, Sjögren's syndrome is manifested by xerostomia and keratoconjunctivitis sicca accompanied by the swelling of the salivary glands. Küttner tumor and Mikulicz's disease are serologically characterized by high levels of serum IgG and its subtype IgG4, and it is mostly negative for the antinuclear antibodies (ANA) and anti-SS-A and -SS-B, which are often positive in Sjögren's syndrome [5]. Histological examination showed the presence of IgG4-positive plasma cell infiltration in Mikulicz's disease but not in Sjögren's syndrome [5]. Chronic sclerosing sialadenitis with widespread involvement of the major and minor salivary glands has been reported as a usual presentation [9]. Mikulicz's disease and Küttner tumor may be subtypes of an identical clinical entity with IgG4-related inflammations, with the extent and severity of the lesions within the salivary gland presenting with different clinical manifestations.

In unilateral cases similar to our tumor-like Cases 2 and 3, it may not be easy to diagnose this disorder. If the submandibular swelling occurs unilaterally with abnormal accumulations on FDG-PET (Case 2), malignancy such as a cancer or lymphoma is more likely to be suspected. Measurement of serum IgG4 concentrations is useful for diagnosing Mikulicz's disease: it was significantly greater in Mikulicz's disease than in other salivary gland diseases (a cutoff value of 135 mg/dL) [10]. It may be also useful in differentiating clinically Küttner tumor from neoplasm. If serum IgG4 concentration is within normal range, biopsy sample should be taken for a definite diagnosis (Case 3). Once a definite diagnosis of IgG 4-related chronic sclerosing sialadenitis is made on the basis of pathology, FDG-PET may be useful for characterizing multifocal lesions other than the salivary gland and/or assessing the therapeutic effects of corticosteroids [7]. Kitagawa et al. [2] found that 5 out of 12 patients with Küttner tumor were associated with sclerosing lesions in the extrasalivary gland tissues. Treatment with steroids shows a good response in Küttner tumor and Mikulicz's disease [3, 5, 11].

In clinical practice, we recommend that Küttner tumor should be treated as a systemic disorder rather than a salivary gland disorder, by otolaryngologists. Clinical whole-body examination and followup is necessary to find simultaneous and/or emerging IgG4-related inflammations of other sites when they did it.

## Figures and Tables

**Table 1:** Summary of three case reports.

	Case 1	Case 2	Case 3
Age, gender	77 years old, male	59 years old, female	61 years old, male
Swelling of the submandibular gland	Both sides	Initially, on the right side; after a period of time, on the left side	Right side
Serum amylase	Within normal range	Within normal range	Within normal range
Serum SS-A antibody	Negative	Negative	Negative
Serum SS-B antibody	Negative	Negative	Negative
Serum IgG (863–1589 mg/dL)	2441	1313	1196
Serum IgG4 (4–108 mg/dL)	451	241	64
IgG4-positive plasma cells in salivary gland tissue	Positive	Positive	Positive
Histopathological diagnosis	Chronic sclerosing sialadenitis	Chronic sclerosing sialadenitis	Chronic sclerosing sialadenitis
Other organ lesions	No	No	No
Other image findings	none	Intense uptake on PET	Decrease in accumulations on salivary gland scintigraphy
